# Molecular Docking and Simulation Analysis of Glioblastoma Cell Surface Receptors and Their Ligands: Identification of Inhibitory Drugs Targeting Fibronectin Ligand to Potentially Halt Glioblastoma Pathogenesis

**DOI:** 10.3390/ijms262010038

**Published:** 2025-10-15

**Authors:** Mohd Wajid Ali Khan, Mohammad Jahoor Alam, Subuhi Sherwani, Sultan Alouffi, Khalid Al-Motair, Saif Khan, Shahper Nazeer Khan

**Affiliations:** 1 Department of Chemistry, College of Sciences, University of Ha’il, Ha’il 2440, Saudi Arabia; 2 Medical and Diagnostic Research Center, University of Ha’il, Ha’il 2440, Saudi Arabia; s.sherwani@uoh.edu.sa (S.S.); k.almutier@uoh.edu.sa (K.A.-M.); 3 Department of Biology, College of Sciences, University of Ha’il, Ha’il 2440, Saudi Arabia; j.alam@uoh.edu.sa; 4 Department of Medical Laboratory Sciences, College of Applied Medical Sciences, University of Ha’il, Ha’il 2440, Saudi Arabia; s.alouffi@uoh.edu.sa; 5 Department of Basic Dental and Medical Sciences, College of Dentistry, University of Ha’il, Ha’il 2440, Saudi Arabia; sf.khan@uoh.edu.sa; 6 Interdisciplinary Nanotechnology Centre, Aligarh Muslim University, Aligarh 202002, UP, India; shahper01@gmail.com; 7 Integral Centre of Excellence for Interdisciplinary Research-4 (ICEIR-4), Department of Biosciences, Faculty of Science, Integral University, Lucknow 226026, India

**Keywords:** glioblastoma, homotypic, heterotypic, molecular interaction, molecular docking

## Abstract

Glioblastoma (GB) is an aggressive brain cancer with high microvascular proliferation. The pathological angiogenesis leads to accelerated tumour invasion and diffused infiltration into the surrounding brain tissues, with a tragically short survival rate. Various transmembrane proteins, which are embedded on the glioblastoma cancer cell surface, interact with diverse extracellular ligands/molecules present in the tumor micro-environment. These ligands play a crucial role in the development, progression, and therapeutic resistance. In the present study, we systematically screened multiple transmembrane protein receptors, and their extracellular ligands involved/implicated in GB cancer cell progression. Additionally, we analyzed the homotypic and heterotypic protein associations within glioblastoma cancer cells to better understand their role in tumor development. Ten well-known and clinically approved GB cancer drugs were selected and retrieved from online databases for molecular docking analyses with extracellular proteins. Among the different ligands analyzed, computational analysis revealed a strong interaction between fibronectin (PDB ID: 3VI4) and the majority of GB surface receptors. Furthermore, molecular docking studies between GB-approved drugs and fibronectin demonstrated the strongest binding interaction with Irinotecan, followed by Etoposide, Vincristine, etc. In conclusion, identification of ligand-drugs interactions provides valuable insights into the mechanisms underlying GB cancer cell development and potential avenues for therapeutic inhibition strategies. Our study demonstrated that Irinotecan, Etoposide, and Vincristine exhibit strong binding interactions with fibronectin, effectively disrupting its interaction with surface receptor(s). Since fibronectin receptor interactions play a crucial role in GB tumor progression, these findings suggest that targeting fibronectin could present a promising strategy to inhibit GB cell proliferation and invasion.

## 1. Introduction

Glioblastoma (GB) is the most common and highly aggressive fast-growing brain tumor. It is characterized by rapid proliferation, high invasiveness, and a grim prognosis, posing numerous challenges in treatment and management of the tumor [1,2]. Despite the advances in surgical techniques, chemotherapeutic, and radiation therapies, the survival rate for glioblastoma patients remains alarmingly low, primarily due to the tumor’s invasive nature, genetic heterogeneity, and the brain’s unique protective barriers which limit the effectiveness of conventional treatments [3]. As a result, ongoing research focuses on progressive strategies to overcome these obstacles, including targeted therapies, immunotherapy, and novel drug delivery systems aimed at improving patient outcomes.

The annual incidence of glioblastoma in the United States underscores the critical need for effective therapeutic approaches. Current research efforts aim to extend the median survival beyond the current 15 months by exploring molecular therapies, immunotherapies, and their integration with conventional treatments [4]. This effort is driven by the tumor’s notorious resistance to standard therapeutic modalities, necessitating the development of novel strategies to improve patient outcomes and overall survival rates.

The biological activities of the tumor microenvironment (TME) cells are also governed by protein–protein interactions (PPIs) [5,6,7]. Approximately 130,000–650,000 different types of PPIs exist in humans [8,9], playing a crucial role in physiological and pathological processes, including cell growth, differentiation, apoptosis, and signal transduction [8,9]. Therefore, due to their pivotal role in disease mechanisms, PPIs represent significant potential in drug discovery and development. Several studies have demonstrated that PPIs participate in disease pathways and modulate disease pathogenesis, making them promising targets for therapeutic interventions [9,10,11].

TME creates environmental conditions that impair the effectiveness of chemotherapy treatment. In addition to tumor cells, the TME consists of various cellular components, including fibroblasts, neural cells, vascular cells, and immune cells [12]. Complex cell–cell interactions within the TME lead to the activation of tumor-induced signals, which alter cellular morphology and functions, ultimately promoting cancer progression. A detrimental cascade of reactions is initiated in the TME, resulting in production of increased levels of inflammatory factors such as growth factors, cytokines, oxidative stress factors, metalloproteinases, etc. These unfavorable secretory molecules disrupt normal cellular signaling pathways, promoting tumor development, tissue invasion, and disease progression [13,14].

Cancer cells communicate with other cells in the TME through cell adhesion molecules that facilitate tumor cell protection and propagation. These interactions are of two types, i.e., homotypic and heterotypic adhesion mechanisms. Homotypic interactions involve the binding of identical proteins on two different cells, whereas heterotypic interactions occur when two different protein molecules interact across cells [15]. Certain regulatory factors, such as L1-cell adhesion molecules, further stabilize these interactions [16]. These cell–cell interactions via adhesion molecules can promote cancer cell migration and metastasis, making them crucial targets for glioblastoma research. Glioblastoma TME and its interaction with diverse cellular components represent a critical area of ongoing research.

Fibronectin is a large dimeric glycoprotein composed of repeating type I, II, and III modules, each harboring distinct binding sites for cell surface receptors and extra cellular matrix [17]. It has long been proposed to play an important role in the pathobiology of cancer and emerging evidence highlights its potential role as a therapeutic target. [17,18,19,20]. Studies have employed either genetic or proteomic approaches to demonstrate aberrant cancerous fibronectin expressions in various types of tumor cells and their experimental and clinical association with tumor malignancy, metastasis, or poor prognosis [18,21,22,23,24,25]. Of particular importance in the progression of GB is the synthesis of key extracellular matrix molecules such as collagen type IV, laminins, and fibronectin by the glioblastoma cells [26]. Tumors exhibit a marked increase in fibronectin levels, which is instrumental in facilitating the invasive migration of glioma cells [27]. Mechanistically, fibronectin stimulates crucial signaling cascades, including the NFκB pathway, thereby fostering cellular proliferation and enhancing resistance to therapy [28]. Research using GL261 glioma models confirms that depleting fibronectin disrupts integrin-mediated signaling, preventing the activation of Src kinase and STAT3 [29]. This interruption subsequently causes a pronounced decrease in the expression of survivin, a vital protein for cell survival [29]. Given fibronectin’s pivotal role in these oncogenic processes, its cellular receptors, integrins, have emerged as highly promising targets for novel GBM interventions, spanning from direct therapeutics and drug delivery systems to imaging-based diagnostics. However, comprehensive studies on molecular interaction of fibronectin with potential ligands remain limited. Additionally, detailed analyses of the interaction of fibronectin with glioblastoma targeting drugs are largely lacking detail. Building on these findings, there is an urgent need to delineate the specific fibronectin–ligand interactions, identify the domains that are most critical, and determine which interactions are “druggable” for effective GBM therapeutic development.

Investigating glioblastoma TME interactions, particularly between glioblastoma cell surface proteins and soluble protein ligands, can provide novel insights into therapeutic targets for drug development. In this study, we employed computational analyses and molecular simulation techniques to screen cell surface proteins on glioblastoma cells and TME-derived soluble protein ligands, assessing their interaction strength and stability. These findings provide a foundation for identifying small molecule inhibitors that can disrupt critical cancer cell interactions. Furthermore, by analyzing the interactions between existing small-molecule drugs and extracellular glioblastoma proteins, we provide mechanistic insights that can inform more effective drug delivery strategies and enhance therapeutic efficacy against glioblastoma.

## 2. Results

In the present study we investigated glioblastoma cancer cell microenvironment by selecting ten different extracellular surface proteins (receptors) along with ten of their associated extracellular matrix proteins (ligand), chosen based on reported involvement in glioblastoma (in various databases and published studies).

### 2.1. Docking Analysis of Targeted Proteins

The three-dimensional structure of glioblastoma cancer cell surface receptors and their corresponding proteins as ligands were retrieved from the PDB database with the following PDB IDs: 3MJG, 1KTZ, 1IVO, 1SHY, 1IAR, 1SUV, 5T89, 1EVT, 3VI4, and 2I9B. These PDB structures were selected due to their high resolution, ensuring more accurate molecular modeling. To prepare the structures for docking simulations, the retrieved PDB structures were optimized and used as receptors. Further, Discovery Studio 2019 suite was used to remove water molecule heteroatoms and add polar hydrogens to the crystal structure in preparation for docking simulations, as mentioned in Table 1.

The molecular docking of the surface receptors (Table 1) and extracellular matrix proteins (Table 2) (homotypic and heterotypic) was performed using the HADDOCK server.

The docking score, rmsd, electrostatic energy, Van der Waals energies, and desolvation energies of all the polypeptide ligands docked against receptor proteins are summarized in Table 3. Moreover, in the present study we focused primarily on the ligand fibronectin (3VI4) due to its high binding energy with the associated protein receptors listed in Table 3. The binding affinity analysis of fibronectin with 10 different glioblastoma surface receptors demonstrated strong interactions with most receptors. A detailed summary of their interactions is provided in Table 3.

### 2.2. Molecular Docking and Molecular Dynamic Simulation Analysis of Surface Receptors Binding vs. Extracellular Fibronectin Ligand

**Figure 1:**
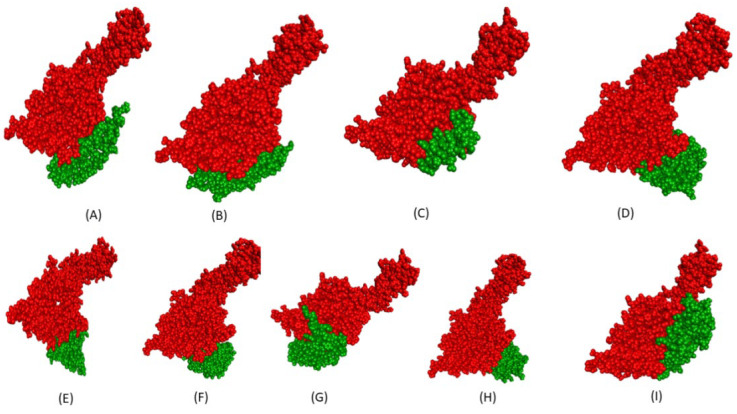
Heterotypic protein–protein (surface receptors vs. extracellular fibronectin ligand) interaction on glioblastoma cancer cell. (**A**) 3VI4-3MJG (3VI4 in red and 3MJG green), (**B**) 3VI4-1KTZ (3VI4 in red and 1KTZ green), (**C**) 3VI4-1IVO (3VI4 in red and 1IVO green), (**D**) 3VI4-1SHY (3VI4 in red and 1SHY green), (**E**) 3VI4-1IAR (3VI4 in red and 1IAR green), (**F**) 3VI4-1SUV (3VI4 in red and 1SUV green), (**G**) 3VI4-5T89 (3VI4 in red and 5T89 green), (**H**) 3VI4-1EVT (3VI4 in red and 1EVT green), (**I**) 3VI4-2I9B (3VI4 in red and 2I9B green).

To validate the molecular interactions between extracellular ligands and their receptors, an in silico study was conducted. In this study, molecular docking simulations were performed to evaluate the binding affinity between glioblastoma surface receptors and extracellular fibronectin ligand (PDB ID: 3VI4). It is well established that more negative docking energy corresponds to stronger binding interactions between proteins and their active targets sites. Molecular docking analysis revealed a relatively strong binding affinity between surface receptors with extracellular fibronectin ligand (−21.3, −18.9, −19.4, −18.5, −17.7, −17.3, −17.7, −20.8, −17.2, and −19.9 kcal/mol, respectively) as detailed in Table 4. These values suggest significant protein–ligand interactions, reinforcing the role of fibronectin in glioblastoma microenvironment modulation. Figure 1 illustrates the heterotypic interactions between surface receptors and extracellular fibronectin ligand in glioblastoma cancer cells, providing a visual representation of these crucial molecular interactions.

**Figure 2:**
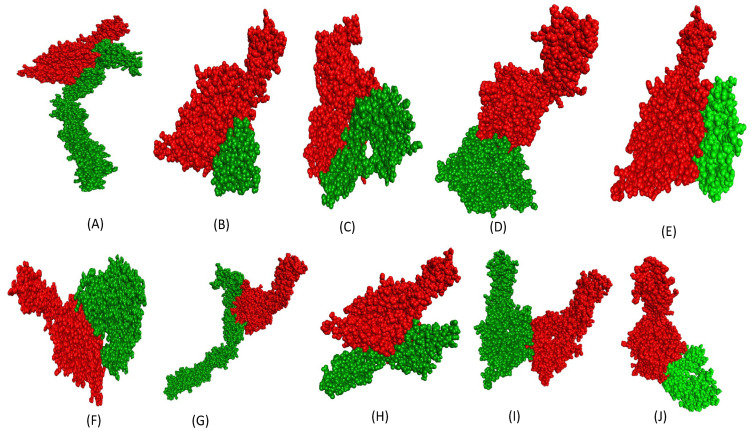
Homotypic protein–protein (surface receptor vs. surface receptor) interaction in glioblastoma cancer cell. (**A**) 3VI4-3MJG (3VI4 in red and 3MJG green), (**B**) 3VI4-1KTZ (3VI4 in red and 1KTZ green), (**C**) 3VI4-1IVO (3VI4 in red and 1IVO green), (**D**) 3VI4-1SHY (3VI4 in red and 1SHY green), (**E**) 3VI4-1IAR (3VI4 in red and 1IAR green), (**F**) 3VI4-1SUV (3VI4 in red and 1SUV green), (**G**) 3VI4-5T89 (3VI4 in red and 5T89 green), (**H**) 3VI4-1EVT (3VI4 in red and 1EVT green), (**I**) 3VI4-3VI4 (3VI4 in red and 3VI4 green), (**J**) 3VI4-2I9B (3VI4 in red and 2I9B green).

Similarly, the homotypic protein–protein interactions were also analyzed for fibronectin surface receptor (PDB ID: 3VI4) vs. other surface receptors (PDB ID: 3MJG, 1KTZ, 1SHY, 1IAR, 1SUV, 5T89, 1EVT, 3VI4, 2I9B) for glioblastoma cancer cells as shown in Figure 2. The binding affinities for the same have been mentioned in Table 5. Molecular docking analysis showed a relatively significant binding affinity between fibronectin surface receptor protein and other surface receptor protein (−19.5, −18.6, −20.4, −18.5, −17.1, −18.5, −16.2, −19.6, −18.2, and −20.8 kcal/mol, respectively) as detailed in Table 5. The representation of binding interactions of receptors is shown in Figure 2.

**Figure 3:**
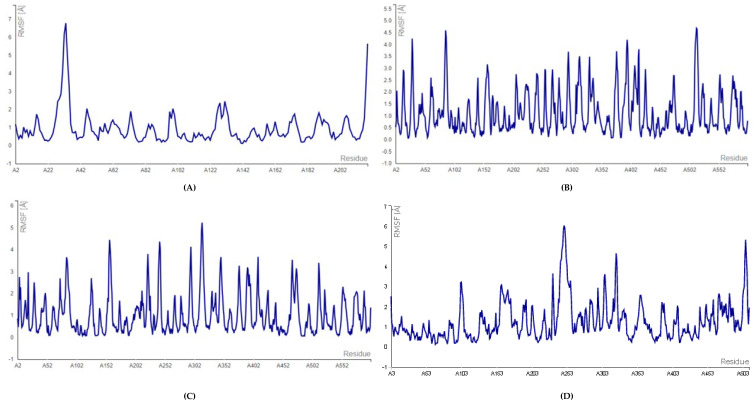

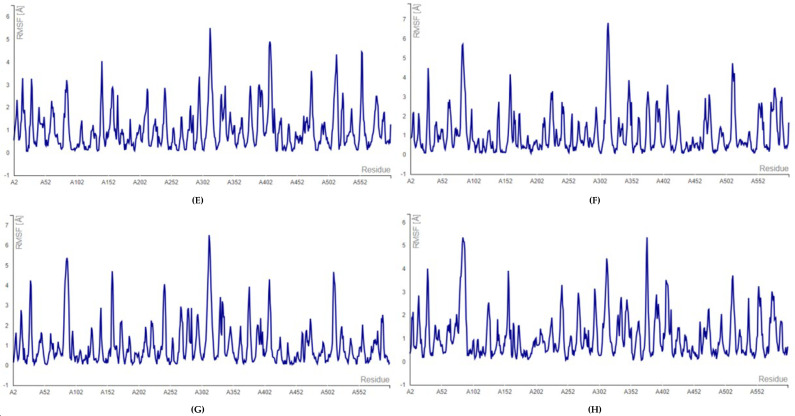

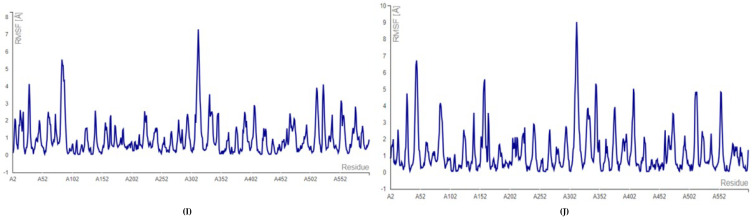
RMSF (root mean square fluctuation) of heterotypic (surface receptors protein vs. extracellular ligand) interactions in glioblastoma cancer cell. (**A**) 3VI4 alone, (**B**) 3VI4−3MJG, (**C**) 3VI4−1KTZ, (**D**) 3VI4−1IVO, (**E**) 3VI4−1SHY, (**F**) 3VI4−1IAR, (**G**) 3VI4−1SUV, (**H**) 3VI4−5T89, (**I**) 3VI4−1EVT, (**J**) 3VI4−2I9B.

To further validate the interaction, we performed molecular dynamics simulations. It was observed that root mean square fluctuation (RMSF) value of 3VI4 (extracellular fibronectin ligand) was lower (1–4 A^0^) when it interacted with surface receptor proteins, which suggested the interaction between surface receptors and extracellular fibronectin ligand (Figure 3) form a stable complex. Similarly, the RMSF value of 3VI4 protein was reduced (0.5–3 A^0^) when interacting with 3VI4 protein. Finally, RMSF value of the surface receptor binding with extracellular ligand protein complex was found to be even lower (0.1–3.5 A^0^), further supporting the stability of the complex.

The RMSF is useful for characterizing local protein mobility in the protein–ligand complex, calculated throughout the simulation. It measures the root mean square displacement of each time residue’s conformation at each time frame relative to the average conformation, providing insight into the flexibility of specific protein regions. In RMSF plot, peaks indicate regions with greater fluctuations during the simulation, while the lower RMSF values reflect areas with less conformational transition. The atomic profile fluctuations were found to be almost similar when comparing the surface receptor binding with extracellular fibronectin ligand (Figure 3). The analysis revealed that the RMSF plot displayed minimal fluctuations in the 3VI4 protein structures. The protein–ligand complexes exhibited higher flexibility at residue number 500, suggesting that the ligand binding site remained relatively rigid throughout the simulation. Further, the contact map of heterotypic (surface receptors vs. extracellular fibronectin ligand) interactions in glioblastoma cancer cells (Figure S1) validates the interaction.

**Figure 4:**
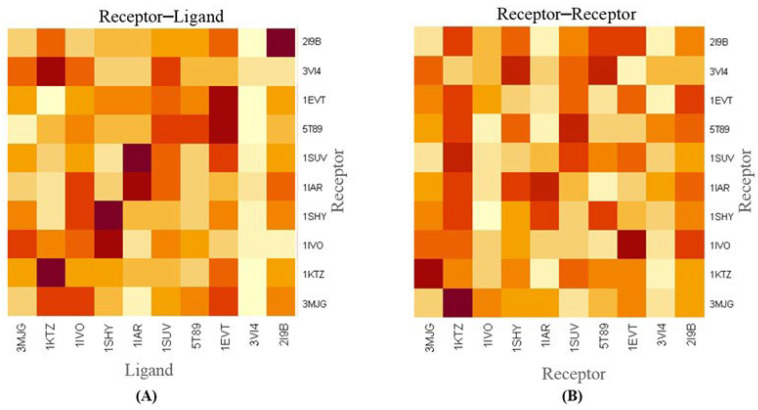
Heatmap showing protein–protein interaction based on binding energy. (**A**) Heterotypic protein−protein (surface receptor vs. extracellular ligand) (**B**) Homotypic protein−protein (surface receptor vs. surface receptor) interaction in glioblastoma cancer cell. Lighter color represents higher binding affinity and darker color represents lower binding affinity. Color intensities from lighter to darker represent higher to lower binding affinities.

In Figure 4, we present the protein–protein association based on binding energy for both heterotypic protein–protein (surface receptor vs. extracellular ligand) and homotypic protein–protein (surface receptor vs. surface receptor) interactions in glioblastoma cancer cells. The heatmap of the surface receptor against the extracellular ligand indicates that binding affinity of the extracellular fibronectin ligand (PDB ID: 3VI4) is higher for most surface receptors (PDB ID: 3MJG, 1KTZ, 1IVO, 1SHY, 1IAR, 1SUV, 5T89, 1EVT, 3VI4, 2I9B), as shown in Figure 4A. Similarly, the analysis of surface receptors vs. surface receptors is presented in Figure 4B. The result highlights the interaction between ligands within the cancer micro-environment and surface receptor–proteins’ interactions in glioblastoma cancer cells.

**Figure 5:**
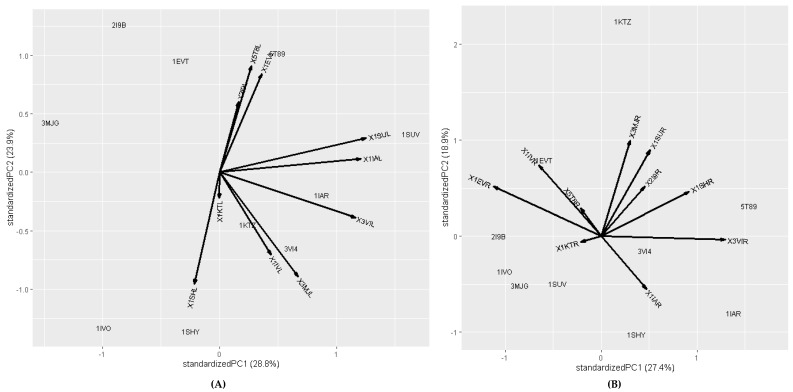
Principal component analysis (**A**) Heterotypic protein−protein (surface receptor vs. extracellular ligand), (**B**) Homotypic protein−protein (surface receptor vs. surface receptor) interactions in glioblastoma cancer cell.

Additionally, the protein–protein interaction based on principal component analysis for both heterotypic (surface receptor vs. extracellular fibronectin ligand) and homotypic protein–protein (surface receptor vs. surface receptor) interactions in glioblastoma cancer cells are shown in Figure 5. Principal component analysis (PCA) plot for receptor and ligand interaction reveals that the first two principal components (PC1 and PC2) represent 52.7% of the total variance within the dataset as shown in Figure 5A. Notably, the first principal component PC1 explained 28.8%, whereas the second principal component PC2 explained 23.9% of the data variation (Figure 5A). Most of the ligands could be included in PC1 coordinates which indicates the higher variability of these extracellular ligands. The extracellular fibronectin ligand, positioned within PC1 coordinates, exhibits higher and positive value, indicating its significant interaction with surface receptor in glioblastoma cancer cells.

Similarly, the PCA plot for surface receptors vs. surface receptors interaction showed that the first two principal components (PC1 and PC2) account for 46.3% of the total variance within the dataset as shown in Figure 5B. The first principal component PC1 explained 27.4%, whereas the second principal component PC2 explained 18.9% of the data variation. Most of the receptors fall within the PC1 coordinates, suggesting higher variability and the formation of stable interactions in glioblastoma cancer cells.

### 2.3. Molecular Docking Analysis (Fibronectin Ligand vs. Drugs)

Fibronectin ligand plays a crucial role in glioblastoma cancer development and progression via signaling cascades including Src kinase and STAT3 signal transduction [17]. Therefore, key cancer drugs (Table 6) have been analyzed as potential blocking molecules for binding of fibronectin ligand to surface receptors.

**Figure 6:**
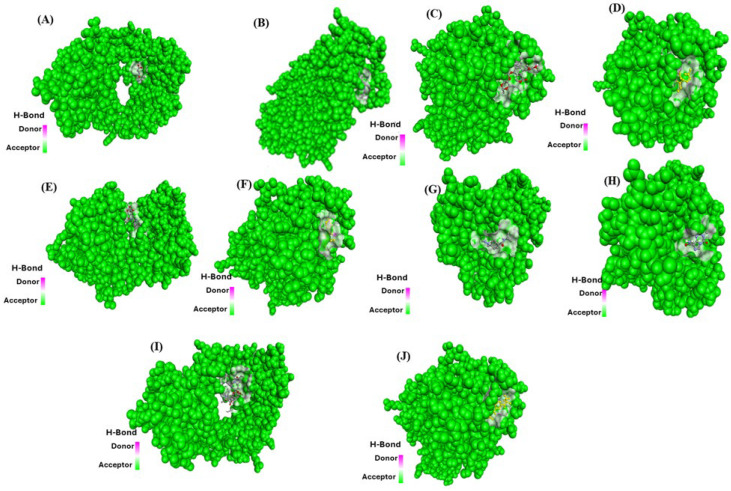
3D structure presentation fibronectin ligand with 10 different drug interactions: (**A**) Fibronectin-5-aminolevulinic acid (fibronectin in green and 5-aminolevulinic acid in violet), (**B**) Fibronectin-carmustine (Fibronectin in green and carmustine in violet), (**C**) Fibronectin-etoposide (Fibronectin in green and etoposide in violet), (**D**) Fibronectin-fluoxetine (Fibronectin in green and fluoxetine in violet), (**E**) Fibronectin-irinotecan (Fibronectin in green and irinotecan in violet), (**F**) Fibronectin-lomustine (Fibronectin in green and lomustine in violet), (**G**) Fibronectin-procarbazine (Fibronectin in green and procarbazine in violet), (**H**) Fibronectin-temozolomide (Fibronectin in green and temozolomide in violet), (**I**) Fibronectin-vincristine (Fibronectin in green and vincristine in violet), (**J**) Fibronectin-5 vorasidenib (Fibronectin in green and vorasidenib in violet).

**Figure 7:**
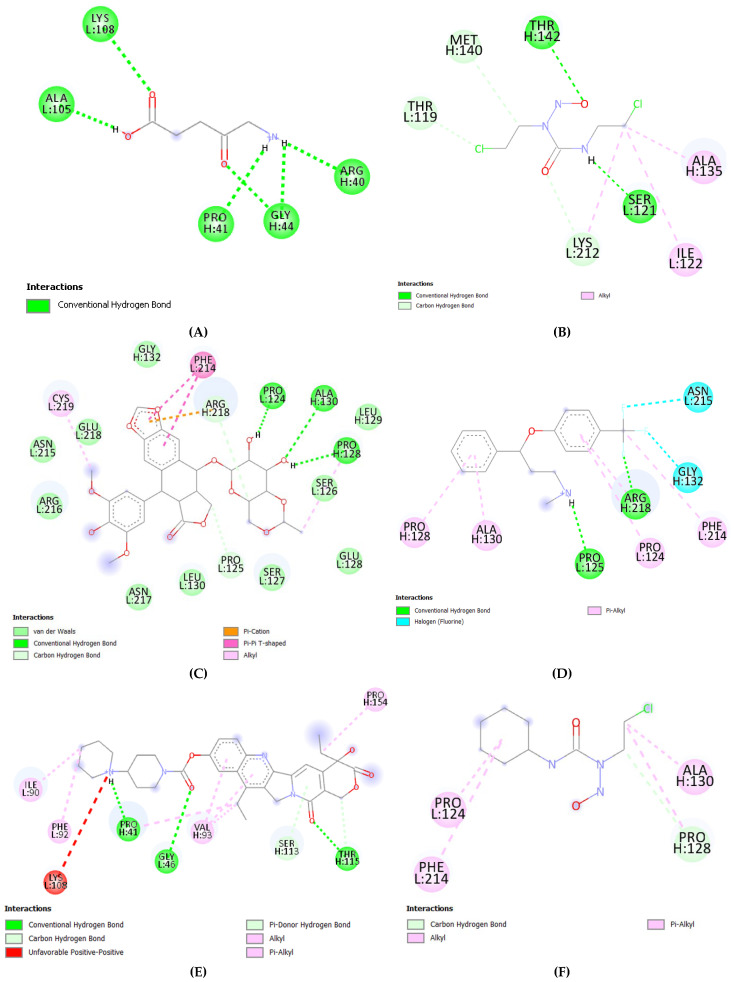

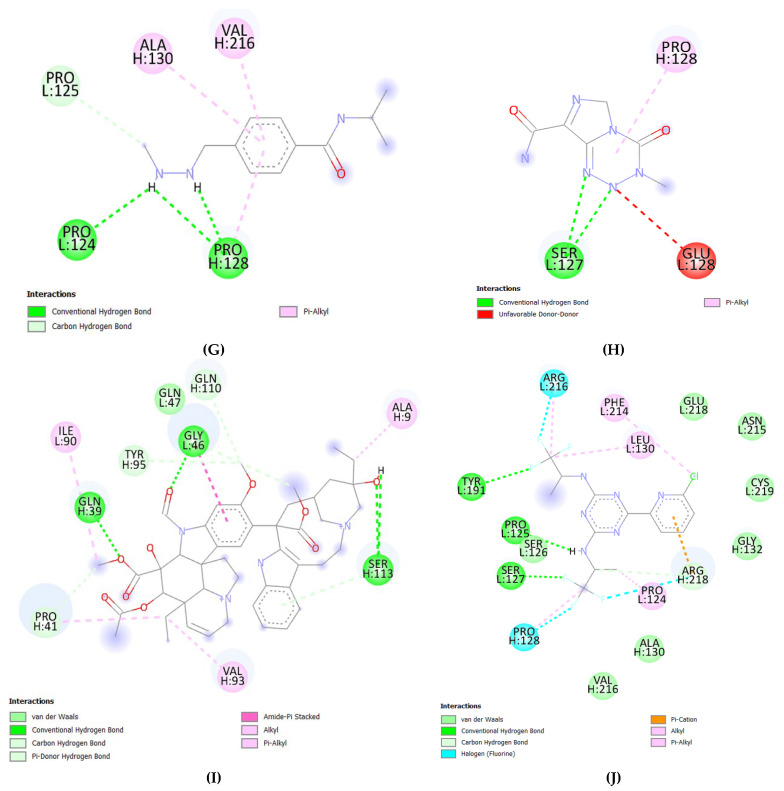
2D structure presentation Fibronectin ligand with 10 different drug interactions: (**A**) Fibronectin-5-aminolevulinic acid, (**B**) Fibronectin-carmustine, (**C**) Fibronectin-etoposide, (**D**) Fibronectin-fluoxetine, (**E**) Fibronectin-irinotecan, (**F**) Fibronectin-lomustine, (**G**) Fibronectin-procarbazine, (**H**) Fibronectin-temozolomide, (**I**) Fibronectin-vincristine, (**J**) Fibronectin-5 vorasidenib.

In the present study, we calculated the binding affinities of complexes formed between the fibronectin ligand and ten selected drugs as shown in Table 4. Results indicate significant binding affinities of all 10 drugs with fibronectin ligand [Figure 6 (3D structure presentation) and Figure 7 (2D structure presentation)]. Our docking studies revealed that out of selected 10 glioblastoma drugs, Irinotecan exhibited the highest binding affinity of −9.4 kcal/mole, while 5-aminolevulinic acid showed the least binding affinity of −4.4 kcal/mole (Table 4).

## 3. Discussion

Glioblastoma is an aggressive and multifactorial malignancy characterized by rapid progression and markedly reduced patient survival [30,31]. Numerous strategies have been designed and developed to create efficient and targeted therapeutics for glioblastoma cells [32]. Effective drug delivery in glioblastoma remains a major challenge due to blood brain barrier (BBB), complex tumor microenvironment, and immune response suppression, etc. [1]. Targeting glioblastoma cell surface receptors and their associated ligands offers a promising approach to disrupt critical processes involved in tumor progression [33]. Several drugs and small molecules have been studied for potential to disrupt signaling pathways for cancer progression, inhibiting tumor growth, and metastasis [33,34]. In the present study, we analysed the binding affinities of glioblastoma cancer cell surface receptors and their associated ligands, focusing on both heterotypic interactions (surface receptors vs. extracellular ligands) and homotypic interactions (surface receptor vs. surface receptor).

Studies on cell surface receptors are currently an important area of research due to their direct involvement in cancer cell signaling and their critical role in tumor progression [31]. Effective treatment strategies should target Ras/RTK/PI3K, pRB, and p53 signaling pathways as well as employ agents such as Bevacizumab against VEGF and DNA alkylating agents (lomustine, carmustine) [35,36].

Molecular docking and simulation approaches were employed to determine optimal molecular interactions between glioblastoma cell surface receptors and their ligands. PDB database was used to retrieve molecular structural data (receptors and ligands) for this study. Molecular docking simulations were applied to evaluate the binding activities between surface receptors and extracellular ligands.

The docking study analyzed multiple parameters, including RMSD, electrostatic energy, Van der Waals energy, and desolvation energy for ligands docked against receptor proteins. Docking score (HADDOCK Score) indicated relatively strong binding affinities between surface receptors and their ligands. Notably, the fibronectin ligand exhibited the highest binding affinity with the majority of surface receptors.

It is well established that more negative docking energies indicate greater binding affinity between the protein and the active site of the target. Molecular docking techniques are therefore pivotal for design and screening of innovative compounds to combat severe diseases afflicting humanity [37,38]. To further confirm protein–ligand interactions, we calculated the root mean square fluctuation (RMSF) of the complexes, as shown in Figure 3. The results further indicate that RMSF values for the protein–protein complex are low, suggesting greater complex stability [39,40]. Additionally, the interaction contact map (Figure S1) highlights significant interaction between the complexes. The contact maps provide a visual representation of the structural basis of protein–protein interactions [41,42]. This also identifies key residues involved in interactions. To assess the significance of interactions, we present a heatmap (Figure 4) of protein–protein across three sections (receptor–ligand, receptor–receptor, and ligand–ligand) with varying colors representing interaction strength (highest to lowest). Furthermore, the principal component analysis (Figure 5) was used to explore and analyze protein–protein interactions [43,44]. The PCA helps reduce data complexity, while explaining the relationships between different proteins. Overall, our analysis reveals that fibronectin (3VI4) is one of the best ligand proteins due to its high binding affinity towards receptor proteins. Based on these findings, we further investigated potential drug delivery strategies in glioblastoma cells, first by characterizing both homotypic and heterotypic protein–protein interactions as described above.

Our results suggest that fibronectin protein could be an important target for drug delivery systems, a finding supported by various studies [45,46,47,48]. We primarily focused on investigating the molecular interactions among extracellular proteins expressed on glioblastoma cancer cells, as reported in previous research and available in public databases [49,50].

Next, we retrieved ten well-known drugs prescribed for GB and docked them against the most promising extracellular matrix protein (fibronectin), based on the results of our protein–protein interaction results. We observed significant binding affinities between the drugs and fibronectin. Notably, some of the commonly prescribed drugs (temozolomide, carmustine, lomustine, and 5-aminolevulinic acid), exhibited comparatively lower binding affinities with fibronectin. This suggests that fibronectin may function as a carrier protein, facilitating drug delivery to the surface of glioblastoma cancer cells.

Despite the valuable computational insights provided by this study, several inherent limitations must be acknowledged. The most significant constraint is the lack of experimental validation, as the proposed drug–fibronectin interactions and their inhibitory effects remain entirely in silico, without confirmation from in vitro or in vivo models that these bindings functionally disrupt fibronectin–receptor signaling and produce the intended anti-tumor effects. Furthermore, simulations employ simplified models that cannot replicate the dynamic complexity of the actual TME, overlooking critical factors like post-translational modifications and competing molecular interactions. From a translational perspective, the study does not address the critical challenge of blood–brain barrier penetration for the identified drugs. Consequently, the proposed therapeutic mechanism whereby drug binding to fibronectin potentially halts tumor progression remains compelling. However, findings in this are preliminary yet provide a strong foundation for future experimental research.

Future prospective studies will extend the current in silico findings by subjecting the identified lead compounds to rigorous in vitro validation to confirm their biological activity. Initial binding and interaction assays, including surface plasmon resonance and ELISA, will be employed to quantitatively demonstrate direct target engagement and dose-dependent disruption of the fibronectin–receptor interaction, with success defined by the identification of inhibitors with nanomolar to low micromolar affinity. Subsequently, various cellular functional assays will determine the phenotypic consequences of this inhibition. This will involve assessing the compound’s ability to impair fibronectin-specific glioblastoma (GBM) cell adhesion, suppress fibronectin-mediated proliferation and viability, and inhibit key pathogenic processes like invasion and migration. The most promising candidate from in vitro screening will then be advanced into an orthotopic xenograft mouse model for the in vivo validation. In this clinically relevant model, luciferase-expressing human GBM cells would be implanted intracranially, and treatment efficacy will be evaluated based on the primary endpoint of overall survival, supported by secondary endpoints such as serial bioluminescence imaging for tumor growth monitoring and histopathological analysis of excised tumors for proliferative index (Ki-67), apoptosis (Cleaved Caspase-3), and target modulation (p-FAK/p-Akt). Concurrently, comprehensive safety and pharmacokinetic/pharmacodynamic studies will be conducted on experimental animals, involving toxicity monitoring of vital organs, blood chemistry, and the critical measurement of drug concentration in brain tissue to confirm adequate penetration and correlate exposure with therapeutic effect.

## 4. Materials and Methods

### 4.1. Retrieval of Protein Receptors

The three-dimensional structures of glioblastoma extracellular protein (beta-type platelet-derived growth factor receptor, TGF-beta type II receptor, epidermal growth factor receptor, hepatocyte growth factor receptor, interleukin-4 receptor alpha chain, transferrin receptor protein 1, vascular endothelial growth factor receptor 1, fibroblast growth factor receptor-1, fibronectin receptor, urokinase plasminogen activator surface receptor), mentioned in Table 1, and associated ligands (platelet-derived growth factor subunit B, transforming growth factor beta-3, epidermal growth factor, hepatocyte growth factor, interleukin-4, serotransferrin, N-lobe, vascular endothelial growth factor A, fibroblast growth factor-1, fibronectin, urokinase plasminogen activator), as mentioned in Table 2, were retrieved from RCSB (https://www.rcsb.org/) database [51] having PDB ID: 3MJG, 1KTZ, 1IVO, 1SHY, 1IAR, 1SUV, 5T89, 1EVT, 3VI4, 2I9B. Refining of the retrieved protein structure was done by removing unnecessary molecules from structure file by using the Discovery Studio suite [52].

### 4.2. Retrieval of Approved Drugs for Glioblastoma

The information of approved drugs was obtained by reviewing various articles available online. Furthermore, we focused on ten well-known and widely used drugs against glioblastoma cancer (as mentioned in Table 3). We retrieved their three-dimensional chemical structures from PubChem database. Furthermore, the retrieved drugs were prepared for molecular docking analysis.

### 4.3. Molecular Docking Analysis

The binding capacity of protein receptors to their target proteins was investigated using molecular docking. For the protein–protein interaction, the HADDOCK-v-2.4 server (https://wenmr.science.uu.nl/haddock2.4/ (02 August 2025)) [53] was used. HADDOCK generates bimolecular complex models and performs protein–protein docking guided by high ambiguity. It incorporates chemical shift perturbation or mutagenesis data associated with biochemical or biophysical interaction data to drive protein–protein docking simulations. The docking process is guided by ambiguous interaction restraints (AIRs) which define ambiguous distances found for all residues that are involved in interaction. The structures were selected based on the lowest intermolecular energies. The docking results obtained from HADDOCK server were further analyzed using CABS-flex (an online tool) (http://biocomp.chem.uw.edu.pl/CABSflex2 (04 August 2025)) [54] for molecular dynamics study of 10 ns to analyze the stability and flexibility of surface receptor binding with vs. extracellular ligands. The results obtained from HADDOCK server were further analyzed using PRODIGY (an online tool) (https://wenmr.science.uu.nl/prodigy/ (5 August 2025)) [55] to determine binding affinity as well as dissociation constant of the protein–protein complex formed by surface receptor binding with extracellular ligand. We performed molecular docking through AutoDockVina tools (version 1.2) to determine the fibronectin–drugs interactions. For ligand binding site of fibronectin, we fixed the parameters of grid box with X = 60, Y = 60, and Z = 60 (center grid box: X = −58, Y = −78, and Z = 39; spacing = 0.347Angstrom) dimensions. The active component and target interaction docking results were exported as pdbqt files and further converted to pdb files by using open babel in the Magica platform. The atomic level structural figures of the docking results were prepared in the chimera software (version 1.19). Further, we visualized the receptor–ligand interaction by Discovery studio 4.0 client.

## 5. Conclusions

Identifying interactions between ligands and drug molecules offers crucial insights into the mechanisms underlying GB cancer cell development and potential avenues for inhibition. Our computational analysis revealed significant protein–protein interaction between transmembrane proteins and extracellular matrix ligand. Screening of ten approved drugs against fibronectin revealed that Irinotecan, Etoposide, and Vincristine exhibit strong binding affinity. Given the critical role of fibronectin–receptor interactions in GB tumor progression, these findings suggest that targeting fibronectin may offer a promising therapeutic strategy to inhibit GB cell proliferation and invasion.

## Figures and Tables

**Table 1:** Glioblastoma cell receptor protein, PDB ID, its chain, and extracellular domains.

S.No.	Receptor	PDB ID	Chain	Extracellular Domain
1.	Beta-type platelet-derived growth factor receptor	3MJG	Chain X, Y	33–532
2.	TGF-beta type II receptor	1KTZ	Chain B	23–166
3.	Epidermal growth factor receptor	1IVO	Chain A, B	25–645
4.	Hepatocyte growth factor receptor	1SHY	Chain B	25–932
5.	Interleukin-4 receptor alpha chain	1IAR	Chain B	26–232
6.	Transferrin receptor protein 1	1SUV	Chain A	89–760
7.	Vascular endothelial growth factor receptor 1	5T89	Chain X, Y	27–758
8.	Fibroblast growth factor receptor-1	1EVT	Chain C	22–376
9.	Fibronectin receptor	3VI4	Chain L, H	21–728
10.	Urokinase plasminogen activator surface receptor	2I9B	Chain E, G	23–305

**Table 2:** Glioblastoma cell receptor’s ligand protein, PDB ID, and its chain.

S.No.	Ligand	PDB ID	Chain
1.	Platelet-derived growth factor subunit B	3MJG	Chain A, B
2.	Transforming growth factor beta 3	1KTZ	Chain A
3.	Epidermal growth factor	1IVO	Chain C
4.	Hepatocyte growth factor	1SHY	Chain A
5.	Interleukin 4	1IAR	Chain B
6.	Serotransferrin, N-lobe	1SUV	Chain C, E
7.	Vascular endothelial growth factor A	5T89	Chain V
8.	Fibroblast growth factor 1	1EVT	Chain A
9.	Fibronectin	3VI4	Chain B, D
10.	Urokinase plasminogen activator	2I9B	Chain A

**Table 3:** Molecular docking analysis along with the energy calculations of protein–protein (receptor vs. ligand) interaction.

S.No	Parameters	3VI4-3MJG	3VI4-1KTZ	3VI4-1IVO	3VI4-1SHY	3VI4-1IAR	3VI4-1SUV	3VI4-5T89	3VI4-1EVT	3VI4-3VI4	3VI4-2I9B
1.	HADDOCK-v.2. Score	−57.3 ± 0.7	80.3 ± 14.2	−24.4 ± 13.3	33.3 ± 2.0	−42.7 ± 4.5	31.8 ± 6.9	47.9 ± 7.3	102.9 ± 3.1	178.4 ± 6.3	186.1 ± 9.9
2.	Cluster Size	43	6	11	80	18	15	25	24	45	6
3.	RMSD from the Overall Lowest Energy Structure	6.6 ± 0.2	0.8 ± 0.5	0.6 ± 0.4	35.3 ± 0.1	1.1 ± 0.6	3.6 ± 0.3	0.7 ± 0.4	13.0 ± 0.1	0.4 ± 0.2	20.3 ± 0.1
4.	Van der Waals Energy	−36.8 ± 4.6	−83.3 ± 13.2	−67.4 ± 8.0	−93.2 ± 4.0	−83.0 ± 7.2	−54.0 ± 2.6	−65.2 ± 8.9	−75.5 ± 5.8	−62.8 ± 9.8	−58.0 ± 4.2
5.	Electrostatic Energy	−115.6 ± 24.8	−224.1 ± 48.0	−332.4 ± 28.1	−222.8 ± 20.8	−271.9 ± 41.7	−500.9 ± 51.5	−401.5 ± 67.8	−299.0 ± 70.1	−711.4 ± 43.0	−432.7 ± 43.8
6.	Desolvation Energy	1.4 ± 0.6	−16.1 ± 3.5	−23.7 ± 3.6	22.5 ± 3.5	−44.1 ± 4.2	23.7 ± 0.6	5.4 ± 4.6	−3.3 ± 5.4	36.0 ± 3.3	16.9 ± 0.6
7.	Restraints Violation Energy	11.8 ± 1.0	2245.6 ± 143.5	1332.8 ± 48.3	1484.8 ± 64.2	1388.4 ± 64.1	1623.2 ± 80.5	1879.9 ± 142.3	2414.5 ± 108.9	3474.8 ± 68.1	3136.9 ± 158.7
8.	Buried Surface Area	1076.7 ± 24.2	2412.0 ± 60.4	2395.7 ± 79.7	2499.1 ± 84.6	2785.3 ± 66.8	2055.4 ± 136.6	2742.9 ± 100.4	2513.0 ± 57.7	2563.2 ± 47.4	2254.6 ± 48.7
9.	Z-Score	−1.2	−1.5	−1.8	−2.2	−2.6	−1.3	−2.1	−1.4	−2.3	−2.5

**Table 4:** Heterotypic interaction between glioblastoma cell extracellular fibronectin ligand vs. surface receptor protein, PDB ID, and binding affinity.

**Ligand**	**Receptor**	**PDB ID**	**Binding Affinity**
Fibronectin (3VI4)	Beta-type platelet-derived growth factor receptor	3MJG	−21.3
	TGF-beta type II receptor	1KTZ	−18.9
	Epidermal growth factor receptor	1IVO	−19.4
	Hepatocyte growth factor receptor	1SHY	−18.5
	Iinterleukin-4 receptor alpha chain	1IAR	−17.7
	Transferrin receptor protein 1	1SUV	−17.3
	Vascular endothelial growth factor receptor 1	5T89	−17.7
	Fibroblast growth factor receptor 1	1EVT	−20.8
	Fibronectin receptor	3VI4	−17.2
	Urokinase plasminogen activator surface receptor	2I9B	−19.9

**Table 5:** Homotypic interaction between glioblastoma cell surface receptor protein vs. surface receptor protein, PDB ID, and binding affinity.

Receptor	Receptor	PDB ID	Binding Affinity
Fibronectin receptor (3VI4)	Beta-type platelet-derived growth factor receptor	3MJG	−19.5
	TGF-beta type II receptor	1KTZ	−18.6
	Epidermal growth factor receptor	1IVO	−20.4
	Hepatocyte growth factor receptor	1SHY	−18.5
	Interleukin-4 receptor alpha chain	1IAR	−17.1
	Transferrin receptor protein 1	1SUV	−18.5
	Vascular endothelial growth factor receptor 1	5T89	−16.2
	Fibroblast growth factor receptor 1	1EVT	−19.6
	Fibronectin receptor	3VI4	−18.2
	Urokinase plasminogen activator surface receptor	2I9B	−20.8

**Table 6:** Molecular docking analysis along with the energy calculations of protein–drugs (Fibronectin ligand vs. drugs) interaction.

S. No	Ligand	Drugs	Binding Energy	RMSD	Image (Drugs)
1.	Fibronectin	Temozolomide	−5.4	1.54	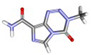
2.		Carmustine	−4.9	1.64	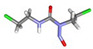
3.		Lomustine	−5.4	1.25	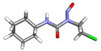
4.		Irinotecan	−9.4	1.81	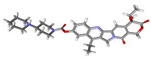
5.		Procarbazine	−5.8	3.22	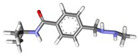
6.		Vincristine	−7.9	2.85	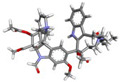
7.		Fluoxetine	−6.2	1.79	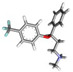
8.		Etoposide	−8.2	0.84	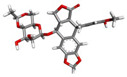
9.		Vorasidenib	−7.9	4.25	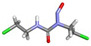
10.		5-Aminolevulinic acid	−4.4	0.64	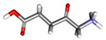

## Data Availability

Data will be available on request.

## Supplementary Materials

The following supporting information can be downloaded at https://www.mdpi.com/article/10.3390/ijms262010038/s1.
