# Supplementary material for: Molecular Docking and Simulation Analysis of Glioblastoma Cell Surface Receptors and Their Ligands: Identification of Inhibitory Drugs Targeting Fibronectin Ligand to Potentially Halt Glioblastoma Pathogenesis
Source: Int J Mol Sci. 2025 Oct 15;26(20):10038. doi: 10.3390/ijms262010038 (PMC12563906; doi:10.3390/ijms262010038)
Supplement: Supplementary file 1 [file ijms-26-10038-s001.zip › ijms-3883429-supplementary.pdf]

Supplementary Figure-S1

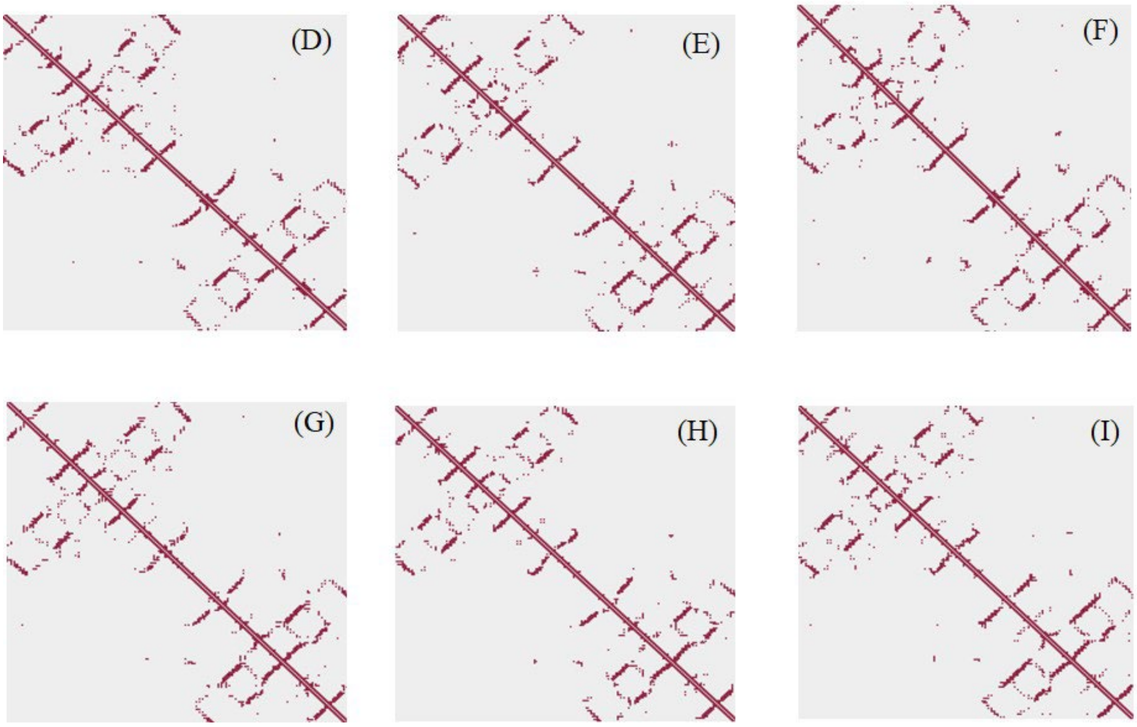

Figure S1: Contact maps of heterotypic protein-protein (surface receptor Vs extracellular ligand) interaction in glioblastoma cancer cells. (A) 3VI4-3MJG, (B) 3VI4-1KTZ, (C) 3VI4-1IVO, (D) 3VI4-1SHY, (E) 3VI4-1IAR, (F) 3VI4-1SUV, (G) 3VI4-5T89, (H) 3VI4-1EVT, (I) 3VI4-2I9B.
